# Role of Uric Acid Levels in the Development of Gestational Diabetes Mellitus: A Review

**DOI:** 10.7759/cureus.31057

**Published:** 2022-11-03

**Authors:** Farhana Yaqoob Khan, Humaira Kauser, Jaimee J Palakeel, Mazin Ali, Sanika Chhabra, Smriti Lamsal Lamichhane, Collins O Opara, Asif Hanif

**Affiliations:** 1 Pathology, Fatima Jinnah Medical University, Lahore, PAK; 2 Medicine, California Institute of Behavioral Neurosciences and Psychology, Fairfield, USA; 3 Neurology, California Institute of Behavioral Neurosciences and Psychology, Fairfield, USA; 4 Tele-geriatric fellowship, Michigan State University, Grand Rapids, USA; 5 General Medicine, California Institute of Behavioral Neurosciences and Psychology, Fairfield, USA; 6 Radiation Medicine, California Institute of Behavioral Neurosciences and Psychology, Fairfield, USA; 7 Public Health, University of Lahore, Lahore, PAK

**Keywords:** elevated uric acid levels, risk factors, hyperuricemia, serum uric acid, predisposing factors, gestational diabetes mellitus

## Abstract

Gestational diabetes mellitus (GDM) is a common disorder affecting pregnancy. Besides conventional risk factors, several novel risk factors have been linked to causing GDM. Increased serum uric acid levels, also termed hyperuricemia, are regarded as one of the significant risk factors for increased insulin resistance and GDM, causing detrimental impacts on both mother and child. The likelihood of developing GDM is at its peak during the first three months of pregnancy in patients with hyperuricemia. Still, its pathophysiology needs to be evaluated in detail. This review is aimed at assessing the function of hyperuricemia in the development of GDM.

## Introduction and background

Gestational diabetes mellitus (GDM) is the most prevalent complication encountered in pregnancy, affecting the health and well-being of millions of pregnant women all across the globe [1]. In 1964, it was formally defined by O’Sullivan and Mahan as high blood glucose levels first diagnosed during pregnancy [2].

It is detected when the pancreas function in females is insufficient to manage the increased diabetogenic effects of pregnancy. Glucose metabolism and tolerance, the sensitivity of peripheral muscles to insulin, and basal glucose synthesis in the liver are normal or somewhat better in early pregnancy. However, maternal insulin sensitivity is decreased due to continual escalation in fetoplacental factors in the mid and late stages of pregnancy. This eventually stimulates maternal cells to utilize fuel sources other than glucose like free fatty acids, which will enhance fetal glucose supply [3]. Even though there is an increase in the number of beta cells and the level of insulin during pregnancy, a number of pregnant females are unable to increase insulin synthesis in response to insulin resistance, thereby developing hyperglycemia and subsequent gestational diabetes [3].

The oral glucose tolerance test (OGTT) is used in the screening of GDM during pregnancy. The primary treatment consists of exercise and dietary control. Pharmacotherapy, including insulin, metformin, and glyburide, can be added. GDM treatment improves pregnancy outcomes [4].

The incidence of diabetes mellitus has been continuously increasing all around the world, reaching the levels of an epidemic. Likewise, the number of pregnant women affected by GDM is also rising, causing an accelerated likelihood of complications in both mother and fetus [4]. According to the report of the International Diabetes Federation (IDF) in 2017, approximately 204 million adult females were living with diabetes, and about 33% of the women were of the reproductive age group. Also, some form of hyperglycemia was found in live births of approximately 21.3 million pregnancies, around 85.1% was due to GDM, and one in seven births was impacted by GDM [5]. As reported by the IDF, the global prevalence of GDM in 2021 was 14.0%, making it a reason for public health concerns [6].

GDM is associated with a number of maternal and fetal complications, including preeclampsia, polyhydramnios, increased odds of cesarean section and induction of labor, congenital malformations, fetal macrosomia, birth asphyxia, respiratory distress syndrome, premature birth, neonatal metabolic complications, neonatal jaundice, and death [1,7]. Various conventional predisposing factors have been pointed out leading to the development of GDM, including maternal obesity, advanced maternal age, family history of type II diabetes mellitus, a previous history of GDM, multiparity, a history of stillbirth or abortion, and polycystic ovarian syndrome [3]. However, several novel risk factors have also been introduced. Several biochemical abnormalities in early pregnancy can lead to the development of GDM at later stages of the pregnancy [8]. Elevated serum uric acid, also known as hyperuricemia, has now been characterized as one of the essential predisposing factors of increased insulin resistance and GDM. Hyperuricemia has been regarded as a marker and predictor for the evolution of diabetes mellitus and metabolic syndrome in the future [9]. It is caused by either increased production or decreased excretion of uric acid. Previous literature has demonstrated that hyperuricemia is linked with obesity, hypertension, hyperinsulinemia, and hyperlipidemia, showing that it could be one component of metabolic syndrome. A study carried out by Kappaganthu et al. demonstrated that elevated serum uric acid levels had a sensitivity of 90%, specificity of 95%, and negative predictive value of 99% for the evolution of GDM in the future [10].

This review is aimed at assessing the function of hyperuricemia in the development of gestational diabetes mellitus.

## Review

Uric acid: clinical characterization and biological importance

Uric acid (C5H4N4O3) is the end product of the catabolism of purines. It is mainly synthesized in the liver, intestine, kidneys, muscles, and vascular endothelium and eliminated by the kidneys and intestines [11]. It exhibits the properties of pro-oxidants and antioxidants. It is responsible for two-thirds of the total antioxidant capacity of plasma. Uric acid is also responsible for the chelation of transition metals [12].

The generation of nitric oxide in endothelial cells is impaired by soluble uric acid, thereby inhibiting the relaxation of vascular endothelium. Thus, increased uric acid levels can cause endothelial dysfunction [13].

In adult men and postmenopausal women, normal serum uric acid level ranges between 3.5 and 7 mg/dL. However in reproductive-age women, the levels of serum uric acid are slightly lower than those in men and postmenopausal women (normal level < 6.0 mg/dL) [14]. An increased glomerular filtration rate and high estrogen levels in premenopausal women are generally related to their lower levels of serum uric acid [15]. It is a significant indicator of insulin resistance, responsible for the development of a metabolic syndrome and type II diabetes mellitus in the future [16].

Hyperuricemia

Hyperuricemia is defined as an increase in levels of uric acid in the serum (>6.8 mg/dL). An increase in the production of uric acid in the liver, inadequate excretion from kidneys and gut, and/or a combination of these two can lead to hyperuricemia [17]. Hyperuricemia is categorized as primary or secondary, relying on its development as an outcome of comorbidity or some medicines [18]. An increase in serum uric acid may occur as a result of the exogenous purine pool and endogenous metabolism of purines. The exogenous pool of purines predominantly depends on dietary intake, especially alcohol, red meat, and sea foods. High sugary and salty foods are also responsible for raising serum uric acid levels. However, the liver, intestines, muscles, kidneys, and vascular endothelium mainly contribute to endogenous uric acid formation. Additionally, the nucleic acids, adenine, and guanine of live and dying cells are degraded into uric acid [19]. Adenine and guanine are converted through deamination and dephosphorylation into inosine and guanosine. Inosine and guanosine are then converted by purine nucleoside phosphorylase into hypoxanthine and guanine respectively, which subsequently are transformed into xanthine by deamination of guanine and xanthine oxidase-oxidation of hypoxanthine. Xanthine oxidase further oxidizes xanthine into uric acid [19].

Various target organs, in addition to the kidneys and joints, are affected by an asymptomatic increase in the metabolism of serum uric acid [20]. Asymptomatic raised serum uric acid levels in adults increase the insulin resistance caused by oxidative stress and inflammatory cytokines, ultimately leading to an increased blood sugar level [21]. An increase in uric acid can be protective, causing opposition to the detrimental impacts of free radical activity and oxidative stress. High levels of serum uric acid can also forecast hypertension development in the future [13]. Increased levels of uric acid in GDM are a major component of metabolic syndrome, reflecting insulin resistance. Clinically, hyperuricemia is regarded as a prognostic marker of kidney disease, metabolic syndrome, cardiovascular disease, and diabetes mellitus, therefore becoming an important predisposing factor for increasing mortality [12]. In the previous literature, hyperuricemia is designated as a predisposing factor to developing insulin resistance and diabetes mellitus within 10 years, predominantly in women [22].

Metabolic syndrome is a combination of various functional and anthropometrical deviations demonstrated by high glucose levels, increased body mass index (BMI), high blood pressure, and abnormally raised lipid profiles. These deviations are also common in patients with hyperuricemia and hyperinsulinemia. It is a major predisposing factor to type II diabetes and various other problems like oxidative stress, mild renal issue, chronic inflammation, and endothelial dysfunction [23]. Hyperuricemia and metabolic syndrome are significantly correlated with high insulin in the blood. A study by Lai et al. showed that there is a mutual inter-related impact of gout and type II diabetes mellitus on raised prevalence. This relationship is complicated but insulin resistance is typically a common connection [24].

Previous literature also indicated that the transfer of amino acids is directly inhibited by hyperuricemia, thereby affecting fetal growth. Also, hyperuricemia causes an increase in the proliferation of endothelium of placental blood vessels, an increase in the production of thromboxane A2 and other vasoconstrictors, and a decline in the formation of nitric oxide, therefore causing small-for-gestational-age babies [25].

Hyperuricemia, insulin resistance, and gestational diabetes mellitus

Increased renal excretion and the uricosuric impact of elevated estrogen levels during pregnancy contribute to decreased levels of serum uric acid [11]. Clearance of uric acid is accelerated in pregnancy, from 6 to 12 mL/min to 12 to 20 mL/min, causing a 25% reduction in blood concentration. It has been suggested that varied renal handling is responsible for causing changes in serum uric acid levels in pregnancy [13]. Various adverse pregnancy outcomes are anticipated if there are high levels of serum uric acid. It may lead to oxidative stress, renal impairment, and cardiovascular disease, which are commonly encountered in severe preeclampsia [26]. Several processes have been proposed to explain the impact of hyperuricemia on pregnancy outcomes. Brien et al. demonstrated discretion in placental system amino acid transfer with hyperuricemia ultimately leading to intrauterine growth restriction. It was also reported that hyperuricemia is capable of causing a dysfunctional placenta. The findings of this study showed that babies born to mothers with hyperuricemia are more prone to perinatal distress [27].

The mechanism of causing insulin resistance in hyperuricemia is the same in both pregnant and non-pregnant females. The sympathetic nervous system may be activated by increased plasma insulin levels, which, in turn, is independently linked to a decreased excretion of uric acid from the kidneys. Increased insulin resistance and altered glucose metabolism in hyperuricemia depend on two hypotheses: 1) through the discretion of endothelial cells’ nitric oxide release (responsible for uptake of glucose to skeletal muscles), or 2) through secretion of uric acid from adipose tissues [28]. A study by Sautin et al. reported that uric acid is responsible for causing endothelial dysfunction and subsequent diminished production of nitric oxide by endothelial cells. Nitric oxide is responsible for insulin’s function on the uptake of glucose in the adipose tissue and the skeletal muscle in animals. Thereby, a reduction in the nitric oxide level causes the diminished uptake of glucose and the resultant development of insulin resistance [29]. The other process in which insulin resistance may be induced by uric acid is that inflammation and oxidative stress in the adipose tissues occur as a result of hyperuricemia, contributing to the development of metabolic syndrome in mice [29].

Acceleration in insulin resistance is anticipated during mid-pregnancy in physiological conditions, which ultimately gets normal after the baby's birth. In mid-pregnancy, several metabolic variations occur enhancing insulin resistance, determined utilizing a homeostatic model for insulin resistance. Maternal obesity, the presence of diabetogenic hormones, and hyperuricemia are the other predisposing factors of insulin resistance during pregnancy [30]. In a study by Weisz et al., it was shown that hyperuricemia and gestational hypertension during pregnancy are significantly associated with increased insulin resistance [31].

The previous literature exhibiting the association of hyperuricemia with GDM demonstrated that hyperuricemia is a significant risk factor for GDM in the first three months of pregnancy [32]. Laughon et al. demonstrated that the likelihood of GDM is elevated 3.25 times in individuals having uric acid ≥ 3.6 mg/dL during the first three months of pregnancy. It was also noticed that the likelihood of hyperuricemia for GDM depends on its concentration (p=0.003) [33]. In another study, it was observed that there is an insignificant association between hyperuricemia and the likelihood of GDM in the second trimester and the postpartum period [34].

GDM is the intolerance of glucose first detected during pregnancy and is responsible for adverse maternal and fetal outcomes if left untreated. GDM is usually initiated in the late second or third trimester of pregnancy and lasts to term. A high blood glucose level usually returns to normal within six weeks of delivery. The worldwide prevalence of hyperglycemia during pregnancy had negative impacts on 16.2% of all live births in the year 2017, with GDM consisting of 86.4% of cases [35].

Hyperuricemia has already been established as an independent predisposing factor for cardiovascular disease, metabolic syndrome, and diabetes mellitus [36]. Hyperuricemia without any symptoms in non-pregnant females enhances insulin resistance caused by oxidative stress and inflammatory cytokines formation, which inexorably raises levels of glucose in the blood [36]. Likewise, it is a significant predisposing factor causing insulin resistance during pregnancy and increases the risk of GDM [36]. High uric acid is related to insulin resistance in pregnancy. So this review focused on the role of hyperuricemia in developing GDM in expecting females.

Several studies have been conducted to assess the association of hyperuricemia with GDM, showing a greater variation in findings. The role of hyperuricemia in predicting GDM is depicted in Table 1.

**Table 1:** The pre- and post-test probabilities of hyperuricemia in the prediction of the development of gestational diabetes mellitus

Study	Sensitivity	Specificity	Positive predictive value	Negative predictive value	Diagnostic accuracy	The uric acid cut-off value
Rehman et al [7]	91.1%	95.7%	86.8%	97.2%	94.5%	-
Kappaganthu et al [10]	90%	95%	-	99%	-	3.4 mg/dL
Sahin et al [16]	100%	60%	-	-	-	3.95 mg/dL
Fawzy et al [37]	77.8%	66.5%	-	-	-	3.15 mg/dL
Chauhan et al [38]	62.5%	99%	-	-	-	>5 mg/dL

Another study reported a significant linear association between serum uric acid concentration in the first 20 weeks and the occurrence of GDM [39]. Ismail et al. also reported that 50-51% of pregnant females with hyperuricemia had GDM [40].

There is a dearth of data as studies report different sensitivities and specificities at different cut-off values [7,16,37]. However, higher accuracies suggest that it can be used in screening gestational diabetes in earlier pregnancy to manage it promptly and accurately later on during pregnancy to avoid unfavorable maternal and neonatal outcomes.

## Conclusions

Insulin resistance due to hyperuricemia has the same course of development and action in pregnant as well as non-pregnant females. Hyperuricemia is associated with an elevated likelihood of developing insulin resistance and gestational diabetes mellitus (GDM), typically by inhibiting endothelial cells to release nitric oxide, mediating inflammatory cytokines, and inducing oxidative variations in adipose tissues by secreting uric acid from adipocytes.

We need further studies to establish a concrete connection between hyperuricemia and GDM. We also need to establish the underlying pathophysiologic mechanisms of this association, as well as the role of different predisposing factors in combination with hyperuricemia in the development of GDM. Further research can assist in early screening and can provide early diagnosis. The resultant prompt treatment of gestational diabetes can prevent the risk of unfavorable outcomes in both the mother and fetus.
